# Active tuberculosis incidence among treatment failure experienced patients in North Wollow Zone: A multicenter historical cohort

**DOI:** 10.1002/hsr2.1997

**Published:** 2024-03-31

**Authors:** Fassikaw Kebede Bizuneh, Seteamlak Adane Masresha, Berihun Mulu Yayeh, Tsehay Kebede Bizuneh

**Affiliations:** ^1^ School of Public Health, College of Health Science Woldia University Woldia Amhara Region Ethiopia; ^2^ Department of Geography and Environmental Studies, Faculty of Social Science Bahir Dare University Bahir Dar North West Ethiopia Ethiopia

**Keywords:** AIDS, Ethiopia, HIV, Northeast, tuberculosis

## Abstract

**Background:**

In Ethiopia, tuberculosis (TB) is a significant cause of death among individuals living with HIV, especially in resource‐limited areas and those who have experienced treatment failure. However, there is the paucity of data regarding TB among treatment failures experienced people living with HIV. This study aimed to estimate the rate and identify predictors of tuberculosis among patients who received second‐line treatment in North Wollo, Northeast Ethiopia.

**Methods:**

A retrospective follow‐up study was conducted on 474 HIV‐infected patients who experienced treatment failure. The study period ranged from January 2015 to September 30, 2021. The incidence of TB was assessed using a Cox proportional hazard regression model, after ensuring that all assumptions were met. Factors associated with active TB were determined by analyzing adjusted hazard ratios and 95% confidence intervals.

**Results:**

In a study of 474 HIV‐positive patients on second‐line antiretroviral treatment, we found an incidence rate of 3.6% with 17 new cases of TB observed over 4412.4 persons per year (PPY). The overall incidence density rate was estimated to be 0.39 cases per 100 PPY (95% CI: 0.239–0.618). Regarding the occurrence of active TB in second‐line patients, WHO clinical treatment stage (T3 and T4), missed isoniazid preventive therapy had a significantly higher risk (AHR: 13.225, 95% CI: 2.894–60.434, *p* = 0.001), while being married was associated with a lower risk (AHR: 0.203, 95% CI: 0.045–0.907, *p* = 0.001).

**Conclusion:**

A high incidence of active TB was observed shortly after initiating second‐line antiretroviral treatment. Factors such as being in the WHO clinical treatment stage (T3 and T4) and marital status were determinants for active TB. To improve overall survival rates, it is vital to enhance early TB screening and implement effective isoniazid preventive therapy.

## Highlighted

- In this study of 474 HIV‐positive patients on second‐line antiretroviral treatment were included.
- Incidence rate of 3.6% with 17 new cases of tuberculosis (TB) observed over 4412.4 persons per year (PPY).
- The overall incidence density rate (IDR) was 0.39 cases per 100 PPY (95% CI: 0.239–0.618).
- Patients with WHO clinical stages III and IV, being married and missed isoniazid preventive therapy has significant implication for action.
- High incidence of active TB observed shortly after initiating second‐line antiretroviral treatment.
- Enhancing early TB screening is significant to improve overall survival rates of cases.

## 1 INTRODUCTION

People living with HIV (PLHIV) are highly vulnerable to TB, a major cause of mortality for this population (1–3). HIV infection greatly increases the risk of active TB, as it impairs cellular immune function and creates a strong synergy with TB. TB is the leading cause of death among PLHIV, highlighting the critical link between HIV infection and TB.^1^, ^2^ The decline in the immune system exacerbates the reactivation of latent tuberculosis bacilli in the lungs. In 2012, out of 8.6 million tuberculosis cases, 13% were HIV positive and in 2014, 12% of the 9.6 million new TB cases occurred in individuals with HIV.^3^, ^4^ According to the WHO 2019 report, TB caused 10.1 million new cases and 1.7 million deaths, making it the leading cause of death from a single infectious agent (9). In Africa, the resurgence of TB is linked to TB‐HIV/AIDS connection and a shortage of skilled healthcare personnel, not control program deterioration^5^, ^6^

In Africa, TB is the second largest disease burden (25%), following Southeast Asia (44%) (12, 13). Additionally, one‐third of HIV‐associated deaths are attributed to TB (9, 11). In 2018, approximately 251,000 deaths occurred among HIV‐infected individuals due to TB, accounting for 33% of all deaths. This rate is significantly higher than the expected case fatality rate of 5% or less as outlined by the WHO.^7^, ^8^, ^9^ The WHO's recent estimation indicates that the risk of developing active TB disease is 21 times higher in HIV‐infected people with a 5‐10% annual risk and 51% lifetime risk of developing active TB than those without HIV.^10^

In 2018, there were approximately 37.9 million new HIV infections worldwide. Sub‐Saharan Africa alone accounted for 75% of the global HIV/AIDS prevalence. By 2019, tuberculosis (TB) had surpassed HIV/AIDS as the leading cause of infectious agent‐related deaths worldwide, with an estimated 10.1 million new cases and 1.7 million fatalities, marking a significant shift before 2020.^11^, ^12^ In sub‐Saharan Africa, incidence rates for children and adolescents are high, with 2,017 cases per 100,000 patient‐years (20). The twin epidemic of TB and HIV caused 0.3 million deaths worldwide in 2017, with a lifetime risk increase from 15% to 22% (1). TB‐associated case mortality accounts for 25%–40% of global deaths and 18%–25% of admissions among PLHIV, despite treatment and detection advances.^13^, ^14^, ^15^

In Ethiopia, 5.9% of HIV‐positive patients newly enrolled in ART in 2016 had active TB (22). The country is among the top 10 TB burden countries, contributing to 87% of global TB cases (23). In sub‐Saharan Africa, 10%–15% of the population is affected by the twin epidemic, with a 51% case fatality rate and higher lifetime risk compared to those without TB and HIV (1, 2). TB‐associated mortality varied by region, with 23.01 cases per 100 person‐years in Tigray for adults^16^ and 17.15 cases per 100 person‐years for children in SNNR regions.^17^ and declining CD4 count is a proxy indicator for treatment failure experienced by patient d.^18^

Co‐infection with TB increases the risk of death in HIV‐positive patients compared to those without TB (5, 16, 25, 26). Scientific evidence has identified baseline socio‐demographic and clinical predictors of TB incidence in HIV‐positive patients, contributing to our understanding of its occurrence.^9^, ^19^, ^20^, ^21^ Despite the effectiveness of antiretroviral treatment in reducing deaths from opportunistic infections in PLHIV, TB remains a significant cause of mortality, responsible for one‐third of deaths among children living with HI.^10^, ^22^ The Ethiopian government aims to reduce TB‐related mortality by 90% and TB incidence by 80% by 2030. However, the high rate of co‐infection continues to pose significant challenges for treatment efforts for treatment failure experienced by patients in Ethiopia.^19^, ^23^

## 2 METHODS

### 2.1 Study setting

This study was conducted in three public hospitals in the North Wollo Zone of the Amhara region in northeast Ethiopia: Tefera Hailu Memorial General Hospital, Woldia Comprehensive Specialized Hospital, and General Teferea Hailu Memorial Hospital. These hospitals were selected due to their early implementation of second‐line antiretroviral therapy (ART) and their catchment population. The study was carried out from February 1 to April 30, 2021. The study specifically focused on HIV‐infected individuals who were on second‐line ART between September 2016 and April 2020.

#### 2.1.1 Study period

The data were collected from patients' charts between February 1 and April 30, 2021.

#### 2.1.2 Study design

A retrospective follow‐up study was conducted in a facility‐based setting.

#### 2.1.3 Source population

The study included all HIV‐infected individuals who received second‐line ART between September 2016 and April 2020.

#### 2.1.4 Inclusion criteria

The study included individuals on second‐line ART and before Second‐line ART started TB cases were excluded.

### 2.2 Sample size determination

The sample size for this study was determined using the double population proportion formula with EPI‐Info software. The variables considered for calculating the sample size were opportunistic infections other than TB. The assumptions used were as follows: P1 (52.1%) represented the percentage of exposed individuals with the outcome, P2 (37.7%) represented the percentage of nonexposed individuals with the outcome, Z α/2 (1.96) corresponded to a 95% confidence interval, and r was set as a 1:1 ratio of nonexposed to exposed. Based on these assumptions, the initial sample size was calculated as 404. Accounting for a 10% nonresponse rate due to incomplete medical records, the final sample size was determined as 445. However, between September 2016 and April 2020, there were a total of 493 HIV‐positive individuals^24^

### 2.3 Dependent variables

The study aimed to assess the incidence of tuberculosis (TB) in individuals receiving second‐line antiretroviral therapy (ART). The outcome of interest was the occurrence of TB, labeled as an “event” (Event = TB or indicated as = 1), while individuals who did not develop TB were considered censored (censor, indicated as = 0). The analysis focused on the duration until TB development after initiating second‐line ART, considering TB occurrence as an event and censoring events such as death, loss to follow‐up, medical transfer, and completion of the observation period.

### 2.4 Independent variable

The study examined several socio‐demographic characteristics, including age, sex, residence, educational status, and marital status. It also considered various clinical and laboratory factors such as WHO clinical stage, CD4 count, viral load, ART regimen, hemoglobin level, functional status, opportunistic infections, ART adherence, co‐trimoxazole preventive therapy (CPT) use, isoniazid preventive therapy (IPT) use, TB contact history, and delays in second‐line therapy as variables of interest.

### 2.5 Operational words

Event: TB infection after second‐line treatment of ART started ART drug adherence: Classified as good (>95%), fair (85‐94%), or poor (<85%) based on the percentage of drug dosage calculated from the total monthly doses of ART drugs. Anemia: Defined as a hemoglobin level ≤10 mg/dL.

### 2.6 Data quality control assurance

Data collection for adults aged 20 years involved categorizing weight status as underweight (<18.5), normal (18.5–24.9), and overweight/obese (≥25.0). For children aged 2–19 years, weight status categories were underweight (below the 5th percentile), normal (5% to <85%), and overweight/obese (85% and above). A standardized and pretested checklist was used to extract information from ART registry forms, follow‐up forms, and medical history sheets. The checklist prioritized relevant variables from national ART formats. It underwent a preliminary review at Woldia Comprehensive Specialized Hospital, with adjustments made based on feedback.^7^, ^25^, ^26^

### 2.7 Data processing and analysis

The data was cleaned, coded, and entered into Epi‐DATA (v4.6.0.2). It was then exported to STATA (v14) for analysis. Statistical summaries and incidence density rates were calculated to assess TB occurrence. The distribution of time‐to‐eventwas examined using Kaplan‐Meier estimates and log‐rank tests to identify significant differences. The Cox proportional hazard assumption was checked through graphical and global tests in the final semi‐parametric regression. Variables with a *p* < 0.25 from the bivariable Cox regression were included in the multivariable analysis. Factors associated with TB incidence were determined based on variables with a *p* < 0.25 in the final Cox regression model, selected using the smallest information criterion value.

## 3 RESULTS

### 3.1 Socio‐demographic characteristics of participants

Out of the total 493 sampled subjects, data were collected from 474 participants, resulting in a card completion rate of 96.15%. The participants had a median age of 41 years with a standard deviation of ±12.48. A significant proportion of the subjects were aged 40 or above (50.2%) and lived in urban areas (60.5%). Nearly half of the participants were married (46.2%), and the majority (91.8%) had disclosed their HIV status (Table 1).

**Table 1:** Socio‐demographic characteristics of HIV‐infected individuals who were on SLART in North Wollo and Waghimira Zone, Northeast Ethiopia, 2021.

Variables	Variable category	Frequency	Percentage
Age in years	<18	42	8.9
	18–40	194	40.9
	41 and above	238	50.2
Sex	Male	260	54.9
	Female	214	45.1
Educational status	No education	216	45.6
	Primary	171	36.1
	Secondary	70	14.8
	Tertiary	17	3.6
Residence	Urban	287	60.5
	Rural	187	39.5
Marital status	Never married	139	29.3
	Married	219	46.2
	Divorced	91	19.2
	Widowed	25	5.3
Religion	Orthodox	390	82.3
	Muslim	80	16.9
	Protestant	4	0.8
Occupational status	Housewife	100	21.1
	Farmer	125	26.4
	Merchant	108	22.8
	Government employee	75	15.8
	Other	66	13.9
BMI	Underweight	185	39.0
	Normal	261	55.1
	Overweight and Obese	28	5.9
HIV disclosure status	Disclosed	435	91.8
	Not disclosed	39	8.2

### 3.2 Clinical characteristics of treatment failure experienced patients

Approximately 92% received PI‐based ART, and 83% had good adherence to SLART. About 17% had a history of OIs during SLART follow‐up, while 85% took cotrimoxazole preventive therapy. The majority (63.3%) underwent at least one session of EAC during second‐line treatment (Table 2).

**Table 2:** Clinical characteristics of HIV‐infected individuals who were on SLART in North Wollo and Waghimira Zone, Northeast Ethiopia, 2021.

Variables	Variable category	Frequency	Percentage
Second line regions	PI‐based	438	92.4
	Non‐PI based	36	7.6
History of regimen substitution	Yes	82	17.3
	No	392	82.7
Drug toxicities	Yes	29	6.1
	No	445	93.9
Drug Adherence	Good	393	82.9
	Moderate	33	7.0
	Poor	48	10.1
WHO treatment stage (T3 and T4)	T1	358	75.5
	T2	70	14.8
	T3 and above	46	9.7
Comorbidity history	Yes	34	7.2
	No	340	92.8
Functional status at SLART	Bedridden	68	14.3
	Working	406	85.7
History of OIS	Yes	79	16.7
	No	395	83.3
IPT status	Yes	252	53.2
	No	222	46.8
CPT's history of taking	Yes	405	85.4
	No	69	14.6
Attrition	Yes	26	5.5
	No	448	94.5
Contraceptive uses after SLART	Yes	169	35.7
	No	305	64.3
History of EAC at second line regimen	Yes	300	63.3
	No	174	36.7

### 3.3 The incidence of TB on second‐line treatment

Out of the 474 HIV/AIDS patients observed for a total of 4412.4 person‐years (PPY), 17 individuals (3.6%) developed active tuberculosis. The overall incidence density rate (IDR) was estimated at 0.39 per 100 PPY, with a 95% confidence interval of 0.239 to 0.618. Since the Kaplan‐Meier survival graph did not cross the half‐life (h1/2) time, the median time to develop active TB could not be calculated.

### 3.4 Survival status of second‐Line ART patients

**Figure 1:**
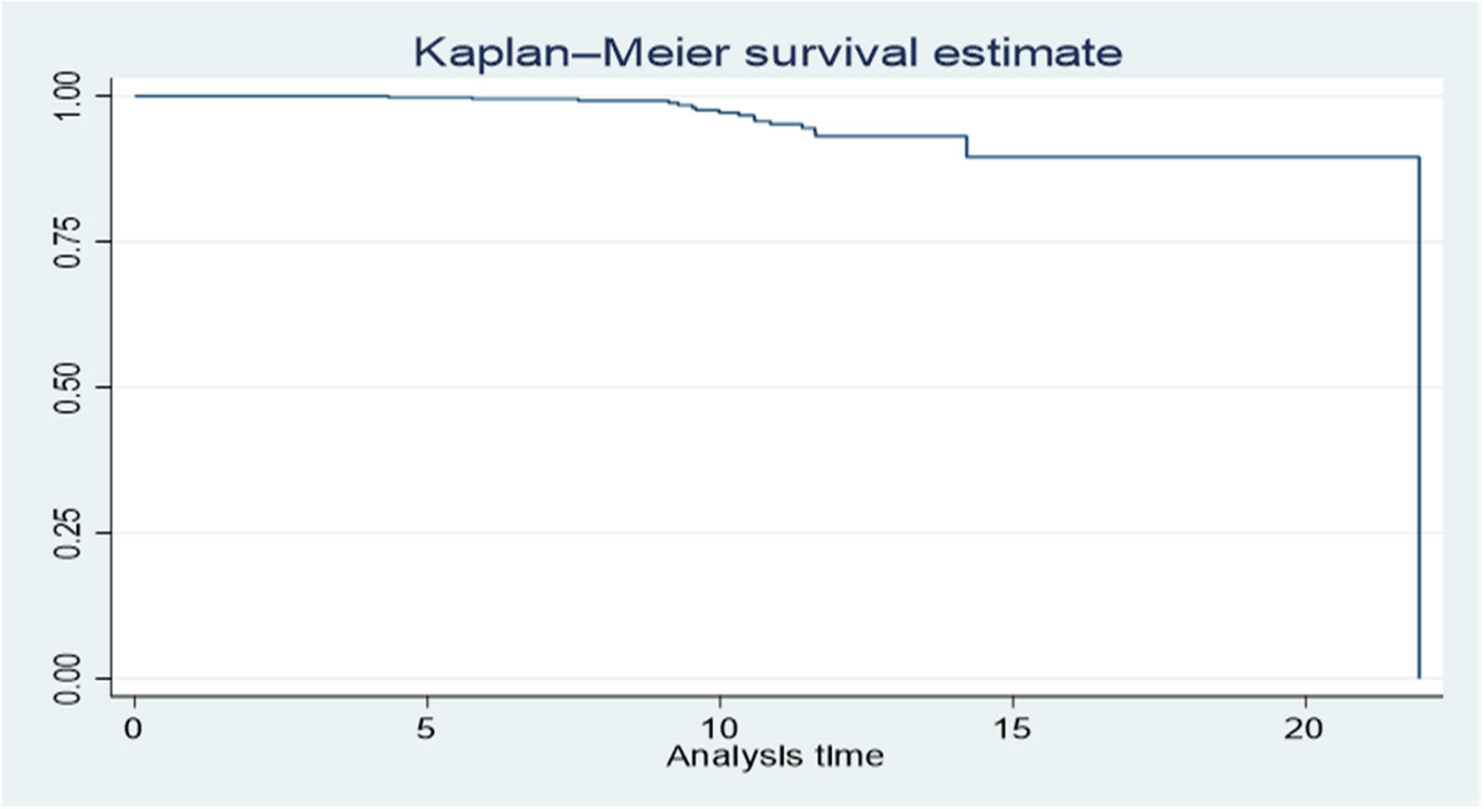
Kaplan‐Meier survival plot for active TB after second line antiretroviral treatment patients.

Significant differences in survival were observed based on marital status and WHO clinical stage (T stage). Among those on second‐line treatment, adherence to isoniazid (INH) during follow‐up had a notable impact on survival. Missing INH significantly affected survival compared to other groups (Chi2 = 56.03, *p* = 0.001). This emphasizes the importance of INH adherence for individuals receiving second‐line treatment. Marital status also influenced survival outcomes. Married individuals had different survival outcomes compared to divorced or widowed individuals (Chi2 (1) = 3.51, *p* = 0.17) (Figure 1). Thus, marital status played a role in determining survival outcomes among individuals undergoing second‐line ART treatment (Figure 1).

**Figure 2:**
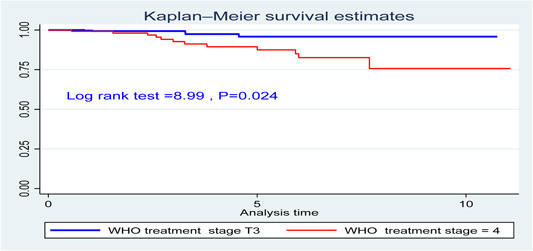
The survival curve difference by WHO treatment stage for active TB incidence and second‐line treatment.

A significant difference in active tuberculosis (TB) development between WHO clinical stages T3 and T4 (chi2(1) = 8.88, *p* = 0.02). Figure 2 illustrates distinct survival patterns for individuals in different WHO clinical stages regarding active TB development (Figure 2).

### 3.5 Factors associated with TB incidence for second‐line treatment cases

Cox regression analyses were performed in this study to identify predictors of tuberculosis (TB) incidence. Several factors were examined, including age, virological failure, residence, functional status, hemoglobin level, isoniazid (INH) use, CD4 count, ART adherence, ART regimen, and counseling during second‐line treatment. The final regression model revealed the following associations with TB incidence:

Patients in WHO treatment stages III and IV had a significantly higher risk of TB incidence (AHR: 13.225, 95% CI: 2.894–60.434, *p* = 0.001).

Missing isoniazid preventive therapy (IPT) after starting second‐line treatment showed a higher risk for active TB, although the association was not statistically significant (AHR: 1.27, 95% CI: 1.345–4.222).

Conversely, individuals who got married after starting second‐line ART treatment had a 79.7% reduced risk of active TB compared to those who were not married (AHR: 0.203, 95% CI: 0.045–0.907, *p* = 0.001) (Table 3).

**Table 3:** Bi‐variable and multivariable Cox‐regression for determinants of active second‐line treatment was initiated in North Wollo selected hospital in Northeast Ethiopia 2023.

Variables	Categories	CHR 95% CI	AHR 95% CI	*p* value
Age	—	0.991 (0.951–1.046)	0.957 (0.889–1.030)	0.934
Virological failure	Yes	1.969 (0.633–6.120)	2.114 (0.432–10.232)	0.242
	No	Ref	Ref	
Marital status	Never married	0.517 (0.151–1.766)	0.139 (0.016–1.233)	0.292
	**Married**	**0.356 (0.113–1.123)**	**0.203 (0.045–0.907)**	**0.078**
	Others	Ref	Ref	
Functional status	Working	Ref	Ref	
	Bedridden	3.148 (1.01–9.816)	1.06 (0.231–4.863)	0.048
**WHO‐ T‐stage**	**T1**	**Ref**	**Ref**	
	**T2**	**1.02 (0.119–8.735)**	0.969 (0.103–9.096)	**0.986**
	**T3 and T4**	**17.018 (5.809**–**49.856)**	**13.225 (2.894–60.434)**	**0.001**
Regiment	PI‐based	Ref	Ref	
	Non‐PI based	2.573 (0.583–11.349)	2.895 (0.331–25.352)	0.212
HIV disclosure status	Not disclosed	2.932 (0.827–10.395)	0.329 (0.027–4.084)	0.096
	Disclosed	Ref	Ref	
Adherence status	Good	Ref	Ref	
	Moderate	2.875 (0.637–12.984)	1.069 (0.096–11.956)	0.170
	Poor	3.134 (0.872–11.261)	1.590 (0.252–10.50)	0.080
IPT	Ever given	Ref	Ref	
	Not given	2.987 (1.037–8.605)	**1.27 (1.345–4.222)**	**0.05**
Council on 2nd ART	Yes	Ref	Ref	
	No	3.078 (1.118–8.476)	1.9 (0.941–5.941)	0.53

*Note*: Others = divorced and widowed.

## 4 DISCUSSION

Among 474 PLHIV with treatment failure, 17 new cases of active TB were reported. The overall incidence rate of active TB was 0.39 cases per 100 PPY, which is lower than previously reported in Addis Ababa,^23^, ^27^ Debre Markose,^28^, ^29^ Afar region,^30^ 7.2 in England.^31^ The difference in protocols for treatment and preventive programs could explain these findings. Additionally, the lower reported incidence of TB could be attributed to variations in follow‐up periods, sample size, and participant characteristics compared to previously reported results.^10^

The inability to calculate the median survival time in this report is attributed to the survival curve not intersecting the median or half‐life point (h1/2). This can be attributed to factors such as insufficient follow‐up time, censoring, low event rate, and limitations in sample size. These challenges make it difficult to accurately estimate the median or half‐life time from the survival curve, often necessitating alternative methods or additional data for more precise estimation^32^ and India.^33^ The difference in outcomes for patients on the second‐line regimen may be due to factors such as CD4 count progression, lack of nutritional support, and treatment adherence issues leading to viral resistance and increased susceptibility to opportunistic infections. Variations in follow‐up periods and sample size may also contribute to the observed differences.

Regarding risk factors for active TB after starting second‐line treatment, a significant association was found with WHO treatment stages III and IV (T‐stage). Patients in this category had a 13.2 times higher risk of developing TB compared to the reference group (AHR = 13.2, 95% CI: 2.894–60.34, *p* = 0.001). This finding is consistent with previous reports in Mekele^32^ and India.^33^ Patients in advanced disease stages (III and IV) may experience weakened immunity, leading to accelerated endogenous reactivation and an increased risk of latent bacilli becoming active TB in the lungs. The compromised immune response associated with advanced disease stages contributes to the development of active TB.^6^, ^34^

Marital status significantly influences the risk of active TB incidence among patients starting second‐line ART treatment. PLHIV living with their spouse on second‐line ART had a 79.7% lower risk of active TB compared to the divorced and widowed groups (AHR = 0.203, 95% CI: 0.045–0.907, *p* = 0.001). This finding is consistent with previous research.^35^ According to previous research findings Married PLHIV in previous research demonstrated higher drug adherence levels compared to single or separated ART users (70.8% vs. 51.6% and 70.8% vs. 66.7%, *p* = 0.009).^36^ The higher ART adherence at 95%, is vital for long‐term effectiveness and preventing drug‐resistant strains. Having a partner fosters social bonding, and support, and encourages ART adherence, leading to improved outcomes. Adequate nutritional support is also crucial for patients on second‐line ART. A partner's presence positively impacts drug adherence by providing affection and care.^37^

The final report of this research indicated treatment failure experienced PLHIV who did not receive isoniazid preventive therapy (IPT) had a significant risk of active TB incidence after treatment failure experienced (AHR: 13.225, 95% CI: 2.894–60.434, *p* = 0.001). This is consistent with previous meta‐analysis findings in Ethiopia^15^, ^18^ and supported with premier studies.^38^, ^39^, ^40^ This might be related to the concomitant IPT with ART administration reducing or demoted new cases of TB incidence and associated deaths by over 90% for PLHIV.^15^ However, factors like IRIS, inadequate active TB screening by a caregiver, low IPT completion of PLHIV, and poor comprehensive care during successive follow‐up shifts end positive outcomes.

## 5 LIMITATIONS OF THE STUDY

A limitation of this study is its retrospective nature, which may introduce biases and limitations in data collection and analysis. Excluding study participants with lost charts could potentially impact the results, especially if these excluded charts were related to TB. This may result in an underrepresentation or incomplete understanding of the relationship between certain factors and TB incidence.

## 6 CONCLUSION AND RECOMMENDATION

A high incidence of active TB was observed shortly after initiating a second‐line antiretroviral treatment. Factors such as being in WHO clinical stages III and IV, as well as marital status, were identified as determinants for the occurrence of active TB. To improve overall survival rates, it is crucial to enhance early TB screening and implement effective isoniazid preventive therapy.

## AUTHOR CONTRIBUTIONS

**Fassikaw Kebede Bizuneh**: Conceptualization; Data curation; Formal analysis; Funding acquisition; Investigation; Methodology; Project administration; Resources; Software; Supervision; Validation; Visualization; Writing—original draft; Writing—review & editing. **Seteamlak Adan Masresha**: Conceptualization; Data curation; Formal analysis; Funding acquisition; Investigation; Methodology; Writing—original draft. **Berihun Mulu Yayeh**: Conceptualization; Data curation; Formal analysis; Funding acquisition; Investigation; Methodology; Project administration; Resources; Validation; Visualization; Writing—original draft; Writing—review & editing. **Tsehay Kebede Bizuneh**: Data curation; Formal analysis; Funding acquisition; Investigation; Methodology; Project administration; Supervision; Validation; Visualization; Writing—original draft; Writing—review & editing.

## CONFLICT OF INTEREST STATEMENT

The authors declare that they have no competing interests.

## ETHICS STATEMENT

The study was conducted following ethical guidelines, including the declaration of Helsinki. The research procedures and objectives were thoroughly reviewed by the institutional review board of Woldia University College of Health Sciences. Ethical approval was granted on February 25, 2022, with reference number RCS, TT & UIL;0015/2015) on 09/12/2015 E.C. Written informed consent was obtained from the legal guardians or next of kin of the patients/participants. For individuals under the age of 18, both parental consent and participant assent were obtained during the early data collection phase.

## TRANSPARENCY STATEMENT

The lead author Fassikaw Kebede affirms that this manuscript is an honest, accurate, and transparent account of the study being reported; that no important aspects of the study have been omitted; and that any discrepancies from the study as planned (and, if relevant, registered) have been explained.

## Data Availability

The full data set used for this manuscript is from the corresponding author upon reasonable request.
